# Compare the prognosis of pancreatic cancer patients with different treatment modalities and use machine learning methods to build predictive models

**DOI:** 10.3389/fmed.2025.1629324

**Published:** 2025-11-26

**Authors:** Lingling Fan, Shujing Kong, Yizhou Deng, Yunfan Wang, Xiancheng Yan, Chao Jiang, Li Tao, Weimin Wang

**Affiliations:** 1 Department of Oncology, Yixing Hospital Affiliated to Medical College of Yangzhou University, Yixing, Jiangsu, China; 2 Department of Pharmacy, School of Medicine, Yangzhou University, Yangzhou, Jiangsu, China; 3 The Key Laboratory of Syndrome Differentiation and Treatment of Gastric Cancer of the State Administration of Traditional Chinese Medicine, College of Medicine, Yangzhou University, Yangzhou, Jiangsu, China; 4 Department of Clinical Medicine, College of Medicine, Yangzhou University, Yangzhou, Jiangsu, China

**Keywords:** pancreatic cancer, multimodal therapies, prognostic markers, machine learning, prediction model

## Abstract

**Background:**

Pancreatic cancer (PC) is highly refractory to most treatments. Multimodal treatment, combining several types of therapies, is likely to benefit PC patients. However, it remains unclear which multimodal treatment is most effective and how to predict outcomes from different combinations. This study compared overall survival among PC patients receiving chemotherapy alone (C), immunotherapy combined with chemotherapy (CI), radiotherapy combined with chemotherapy (CR), and triple-combination therapy (CRI). A machine learning-based predictive model between monomodal and multimodal therapy was established using 3 years of clinical follow-up data.

**Methods:**

We retrospectively analyzed 125 cases of PC patients treated at Yixing People’s Hospital from January 2014 to June 2024 (C, *n* = 50; CI, *n* = 38; CR, *n* = 18; CRI, *n* = 19). The group CI, CR and CRI were merged and defined as multiple modalities (MM) group (*n* = 75), while the group C was defined as single modality (SM) treatment group (*n* = 50). Kaplan-Meier plots estimated the overall survival rate of each group and the survival rate of the SM group and the MM group. Cox proportional hazard models identified key prognostic factors, including cytokines and inflammation mediators. Four machine learning models, including logistic regression (LR), support vector machine (SVM), random forest (RF), and Extreme Gradient Boosting (XGBoost) were used to build predictive models. SHapley Additive exPlanations (SHAP) identified significant contributors to treatment outcomes.

**Results:**

Multimodal treatments significantly improved PC prognosis (*P* = 0.0025). Univariate and multivariate Cox regression analysis showed that interleukin-2 (IL-2) was a protective factor, while neutrophil-to-lymphocyte ratio (NLR) was a risk factor. This study evaluated and compared the predictive performance of four machine learning models using the classifiers such as area under curve (AUC), accuracy and F1 score, etc. In the binary classification task, RF and XGBoost models both achieved good performance compared with the other two machine learning methods. In addition, SHAP analysis also proved that IL-6 contributed the most to the machine learning models.

**Conclusion:**

PC patients may benefit from more intensive multimodal therapies, which provides novel insights into predicting PC survival prognosis and highlights the potential of machine learning in biomarker identification and disease prognosis.

## 1 Introduction

Pancreatic cancer is a highly fatal malignancy, often diagnosed at an advanced stage with limited treatment efficacy (1). Despite recent therapeutic advancements, the prognosis for pancreatic cancer remains poor, highlighting the urgent need to develop new biomarkers and predictive models for early diagnosis, precise treatment, and survival prediction (2, 3). The early diagnosis of pancreatic cancer usually relied on imaging and hematological indicators, but the sensitivity and specificity of these methods were limited, and it was difficult to meet clinical needs (4). Moreover, pancreatic cancer patients exhibited variable responses to treatment, and there was a current lack of prognostic indicators to predict treatment outcomes (5, 6). Therefore, the search for new biomarkers and the use of computer science methods for comprehensive analysis has become one of the hot spots of pancreatic cancer research.

In the treatment of pancreatic cancer, combined treatment modalities have been shown to be superior to single treatment approaches with significantly improved survival rates, enhanced disease control rates, as well as reduced tumor burden. A systematic review and network meta-analysis showed that combined chemotherapy regimens based on gemcitabine or 5-FU were effective in advanced pancreatic cancer, especially the regimen combining gemcitabine with 5-FU derivatives, which was superior to the regimen combined with platinum drugs (7). A single-center study on elderly patients with advanced pancreatic cancer found that the median overall survival in the combined chemotherapy group was 8.2 months, significantly higher than the 4.7 months in the single-drug chemotherapy group, and the median progression-free survival of the combined chemotherapy group was longer (8). However, clinical outcomes remain highly heterogeneous, and it is unclear which patients derive the most benefit from each therapeutic approach. Moreover, the underlying molecular determinants of treatment sensitivity remain largely unclear, posing a major challenge for optimizing therapeutic strategies in pancreatic cancer.

In recent years, with the advancement of molecular biology technology and the development of data science, the application of machine learning in the medical field has gradually increased. Machine learning can mine potential rules from a large number of medical data, and help clinicians make more accurate decisions by building predictive models, especially in the early diagnosis of diseases, prognosis assessment and individualized development of treatment plans. In particular, machine learning algorithms such as Support Vector Machines (SVM), Random Forest (RF), and XGBoost had demonstrated strong performance in processing medical data due to their efficiency, accuracy, and robust non-linear fitting capabilities (9).

Immunoinflammatory factors played an important role in the prognosis of pancreatic cancer, with their levels fluctuating according to treatment and disease progression (10, 11). Dynamic monitoring of these factors can enhance prognostic accuracy. Elevated levels of pro-inflammatory and immunosuppressive factors were generally associated with poorer outcomes, while anti-tumor immune responses correlate with better prognosis (12). For instance, IL-2 has been shown to promote antitumor immunity by activating cytotoxic T lymphocytes and NK cells, and high IL-2 levels are generally associated with favorable outcomes in several malignancies. At the same time, IL-2 also drives expansion of regulatory T-cells (Tregs) and can lead to effector T cell exhaustion or toxicities, thus limiting its therapeutic benefit (13). In pancreatic ductal adenocarcinoma (PDAC), elevated IL-2 levels are associated with better prognosis and enhanced antitumor immunity, and that IL-2-based co-culture with human peripheral blood mononuclear cells (PBMCs) significantly improves dendritic cell (DC) tumor infiltration and T-cell activation, providing a promising strategy to optimize DC-based immunotherapy for PDAC (14). IL-4 and IL-13 are reported to promote pancreatic cancer progression *via* Type II IL-4 receptor signaling, which enhances tumor proliferation, invasion, and immune escape in pancreatic cancer (15). IL-6 family plays a central role in sustaining this pro-tumorigenic inflammation. Accumulating evidence indicates that IL-6 signaling not only enhances cancer cell proliferation and survival but also contributes to stromal activation and immune suppression (16). Preclinical studies indicate that targeted inhibition of IL-6 may enhance the efficacy of anti-PD-L1 in PDAC (17). IL-17A and IL-17B were two subtypes closely related to pancreatic cancer in the IL-17 family (18). IL-17B enhanced the invasion and metastasis ability of pancreatic cancer cells by activating IL-17RB and its downstream ERK1/2 signaling pathway (19). In pancreatic cancer, the expression level of interferon gamma (IFN-γ)-related genes was closely related to the patient’s prognosis. Highly expressed IFN-γ-related genes (such as STAT1) were associated with disease-specific survival (DSS) and extended total survival (OS) of pancreatic cancer patients (20). The NLR, or neutrophil-to-lymphocyte ratio could be an independent indicator of poor prognosis in patients with unresectable pancreatic cancer (21). Combining multiple immunoinflammatory factors (e.g., IL-2, IL-4) with NLR can improve prognostic prediction accuracy (22).

In addition, many clinical studies had shown that β2 microglobulin (β2-MG), lactate dehydrogenase (LDH) played an important role in predicting the prognosis of pancreatic cancer. β2-MG was a marker of various tumors, and the increase in its level was usually associated with an increase in tumor load and poor prognosis. In colorectal cancer, low β2-MG mRNA expression was a powerful predictor of lymph node metastasis and/or poor prognosis (23). While there is limited evidence supporting β2-MG as a standalone prognostic marker for pancreatic cancer, its combination with serological markers, such as high preoperative levels of serum tumor markers such as glycan carbohydrate antigen 19-9 (CA19-9), carcinoembryonic antigen (CEA), and cancer antigen 125 (CA125), is associated with worse tumor differentiation and shorter overall survival. LDH has been generally considered to be a sign of high tumor burden, and the increase in its level was also associated with a high risk of solid tumor death (24). Therefore, how to comprehensively analyze the expression information of different biomarkers through machine learning technology and build an efficient survival prediction model has become an important direction of pancreatic cancer prognosis research.

This study aims to explore the prognostic biomarkers of pancreatic cancer patients by using clinical data such as immune factors and hematological indicators, combined with machine learning algorithms. We used four commonly used machine learning models-LR, SVM, RF, and XGBoost to predict patient survival, and based on these models assessed the impact of different treatment modalities on patient survival. By constructing accurate prognostic models, we aim to provide clinicians with personalized treatment plans and a robust foundation for future research.

## 2 Data and methods

### 2.1 Study population

The Ethics Committee of Yixing People’s Hospital approved the study (Approval No. 2025科085-01), which was in accordance with the Declaration of Helsinki (Revised 2013). A total of 177 PC patients hospitalized in the Department of Oncology of Yixing People’s Hospital from January 2014 to June 2024, were included in the study. The inclusion criteria for this study were: (1) cytologically or histologically confirmed metastatic pancreatic cancer; (2) The chemotherapy regimens for the four groups were limited to AG or FOLFIRINOX, immunotherapy was limited to PD-1/PD-L1 inhibitors, and radiotherapy was limited to intensity-modulated radiotherapy. Among them, immunotherapy was administered concurrently with chemotherapy; the treatment sequence was first chemotherapy (regardless of whether combined with immunotherapy), followed by radiotherapy; the treatment cycle and dose were both based on the CSCO Pancreatic Cancer Diagnosis and Treatment Guidelines; (3) patients with complete clinical information and available cutoff points for recurrence and mortality; and (4) patients without severe infections, autoimmune diseases, and other comorbidities. Patients will be excluded from the study if they have severe cardiac, hepatic and renal comorbidities or incomplete medical data (more than 20% missing data), who were not treated according to the prescribed treatment plan or patients with multiple primary cancers.

### 2.2 Data collection and analysis

Baseline data of all patients were retrieved and recorded from the hospital information system (HIS), including basic information such as age, gender, and tumor histochemical type; inflammation-related markers such as IL-2, IL-4, IL-6, IL-17, and IFN-γ in the first visit; and tumor markers such as CA19-9, CEA, alpha-fetoprotein (AFP); serum biochemical markers such as NLR, LDH, β2-MG levels. The follow-up endpoint was OS, defined as the duration from the patient’s first treatment to death or making the last follow-up date, with a follow-up cut-off date of September 23, 2024. Baseline data including 14 variables were included in the statistical analysis (Table 1). We divided the population into two groups based on the age cutoff of 65, with one group being those aged 65 and above and the other being those aged below 65. The CI (chemotherapy + immunotherapy), CR (chemotherapy + radiotherapy), and CRI (chemotherapy + radiotherapy + immunotherapy) groups were merged to form the Multiple Modalities (MM) group (*n* = 75), whereas the group C (chemotherapy alone) was designated as the Single Modality (SM) treatment group (*n* = 50). Numerical differences between two groups were assessed by the Chi-square test or Fisher’s exact test for categorical variables, while the *t*-test and Kruskal-Wallis H test or Mann-Whitney U test were used for continuous variables. The threshold for significance was *P* = 0.05. Data analyses were performed using Python, Version 3.8.8.

**TABLE 1:** Baseline demographic and clinical characteristics.

Factors	SM (*n* = 50)	MM (*n* = 75)	*P*	*SMD*
Gender, *n* (%)			1.000	0.034
Female	30(60%)	40(53.33%)		
Male	20(40%)	35(46.67%)		
Age (years), *n* (%)			0.879	0.132
≥65	9(18%)	25(33.33%)		
<65	41(82%)	50(66.67%)		
Histologic type, *n* (%)				
Adenocarcinoma	35(70%)	40(53.33%)	0.924	0.126
Non-adenocarcinoma	15(30%)	35(46.67%)		
Laboratory test, median (IQR)				
IL-2 (ng/L)	1.66 ± 1.25	1.69 ± 1.30	0.922	0.018
IL-4 (ng/L)	3.47 ± 2.34	4.12 ± 3.40	0.202	0.225
IFN-γ (U/L)	22.57 ± 20.83	22.22 ± 24.08	0.930	0.016
IL-6 (ng/L)	54.68 ± 96.51	135.10 ± 358.04	0.066	0.307
IL-17 (ng/L)	43.66 ± 36.62	31.68 ± 28.48	0.052	0.365
NLR	0.66 ± 0.14	0.69 ± 0.14	0.160	0.257
LDH (IU/L)	235.07 ± 184.82	223.97 ± 175.05	0.736	0.062
β2-MG (mg/L)	2.61 ± 2.15	3.30 ± 1.56	0.053	0.013
CEA (ng/mL)	32.15 ± 77.90	30.86 ± 116.28	0.941	0.138
AFP (ng/mL)	3.00 ± 1.83	2.73 ± 2.06	0.443	0.074
CA19-9 (U/L)	2201.76 ± 7535.19	1728.18 ± 5024.37	0.695	0.366

As shown in Table 1, baseline characteristics between the monomodal and multimodal groups were compared using both traditional hypotheses testing by calculating *P*-values and standardized mean differences (SMDs). Reporting both *P*-values and SMDs is beneficial because they provide different but complementary information about research results. *P*-values assess statistical significance, indicating the likelihood of an observed difference being due to chance. There were no significant differences in inflammatory-related markers, tumor markers, and serum biochemical markers between the two groups. All *P*-values were greater than 0.05. SMDs, on the other hand, quantify the size of the effect, independent of sample size. Ideally, an SMD value < 0.1 is considered a small difference, an SMD > 0.1 and ≤ 0.2 is a moderate difference, and an SMD > 0.2 is a substantial difference (25). In some cases (IL-4, IL-6, IL-17, NLR and CA19-9), a result with a *P*-value > 0.05 and an SMD > 0.2 indicates that while the difference between two groups is not statistically significant, it may still represent a small to moderate effect that is practically important. This situation often arises due to an insufficient sample size.

### 2.3 Prediction model construction and validation

To analyze prognostic biomarkers and elucidate the relationship between different treatment modalities and survival in pancreatic cancer patients, four common machine learning algorithms were used. Initially, LR was chosen as the baseline model to explore the relationship between biomarkers and patient survival using its linear nature. As a traditional classification algorithm, logistic regression is suitable for dealing with medical data with binary classification problems and is able to quantify the importance of features by estimating regression coefficients. Secondly, SVM is applied to establish decision boundaries. SVM maximizes the classification interval by finding the optimal hyperplane, and is able to effectively deal with non-linear relationships between features and adapt to complex patterns in pancreatic cancer prognostic data. Additionally, RF algorithm, as an integrated learning method, improves the stability and robustness of the model by integrating multiple decision trees to reduce overfitting. Each decision tree is trained on a subset of the data, and the final prediction is made by voting, which makes the random forest better able to cope with high-dimensional feature data. Lastly, XGBoost, an advanced Boosting Tree algorithm, was implemented. XGBoost iteratively trains the model and corrects errors from previous rounds, thereby significantly improving accuracy and generalization. It is particularly adept at handling large-scale datasets and exhibits strong resistance to noise.

These algorithms were chosen based on their general application to medical data and their ability to handle high-dimensional data and classification problems. By training these four models on the same dataset, it is possible to provide diverse solutions for prognostic prediction of clinical pancreatic cancer patients. We used PyCharm (version 3.8.10), combined pandas, numpy, scikit-learn, imbalanced-learn, matplotlib and other common libraries for four machines The learning model (LR, SVM, RF, XGBoost) was modeled and evaluated, among which the XGBoost model additionally used the XGBoost library. To alleviate the class imbalance problem, we uniformly use the SMOTE method from the imbalanced-learn library to resample the training data. The modeling process uses scikit-learn pipeline, which integrates normalization processing and classifier construction. In order to minimize the impact of uneven data distribution on the model evaluation results, the stability and accuracy of the generalization ability assessment of the model are improved by randomly splitting the overall dataset into training and test sets with a ratio of 7:3. Models are fitted on the training set and their generalization performance is evaluated on an internal independent test set.

### 2.4 Model evaluation

In order to comprehensively assess the performance of each machine learning model in prognosis prediction of pancreatic cancer patients, multiple evaluation metrics were used. These metrics can reflect the accuracy and stability of the models from different perspectives. The receiver operating characteristic (ROC) curve and area under curve (AUC) value were employed as critical evaluation standards. We also employed accuracy, the most commonly used metric for classification model evaluation, measured the proportion of correct predictions made by the model. To integrate Precision and Recall, the F1 Score was used as a balanced evaluation criterion. It represents the harmonic mean of precision and recall and is especially suitable for imbalanced classification problems. Model evaluation indicators include AUC, ROC, Accuracy, Specificity, Sensitivity (Recall), Precision, F1 Score, Average Precision (AP). Model evaluation indicators were provided and implemented by scikit-learn. Model visualization, including ROC, Precision-Recall (PR) curves and confusion matrix diagrams were drawn by matplotlib.

### 2.5 Model interpretability

Interpretability of machine learning models was crucial in medical research because it not only helped to improve the transparency of the model, but also helped clinicians understand the basis of the model’s predictions. This was particularly important in prognostic analyses of complex diseases like pancreatic cancer, where explaining the model’s decision-making process is crucial for clinical application. SHAP values (Shapley Additive Explanations), an interpretability method based on game theory, were employed to quantify the contribution of each feature to individual predicted outcomes. SHAP values reveal the positive or negative impact of features on model predictions, thereby clarifying their role in different outcomes. The SHAP package was used to analyze the interpretability of each model, in which LR, RF, and XGBoost generated a summary chart of SHAP values and a bar chart of the importance of features.

**FIGURE 1:**
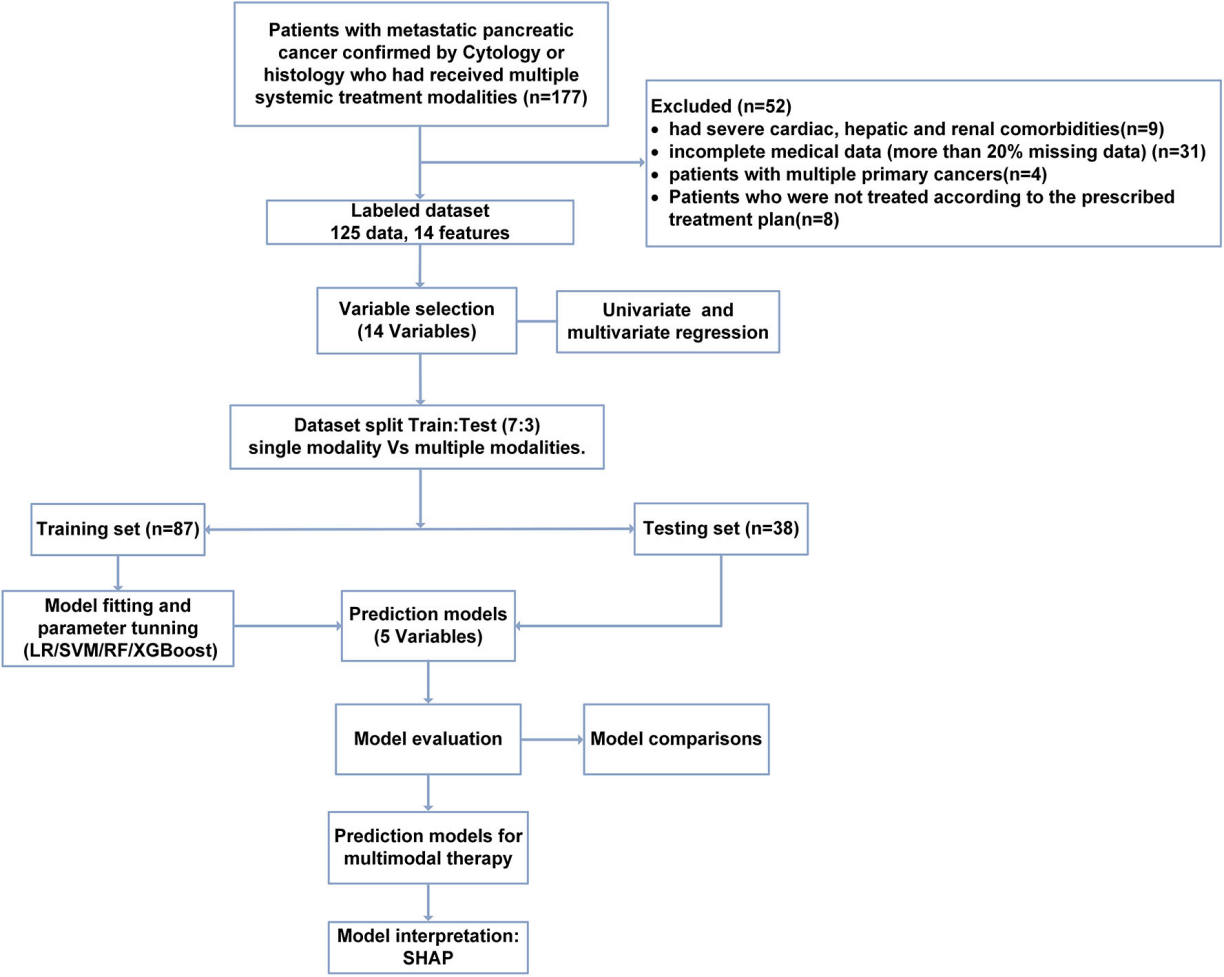
Flow chart of study population selection and model construction.

The data collection, model construction, and evaluation processes are shown in Figure 1.

## 3 Results

### 3.1 The prognosis of multimodal combined treatment for patients with advanced pancreatic cancer is significantly better than that of monomodal.

**FIGURE 2:**
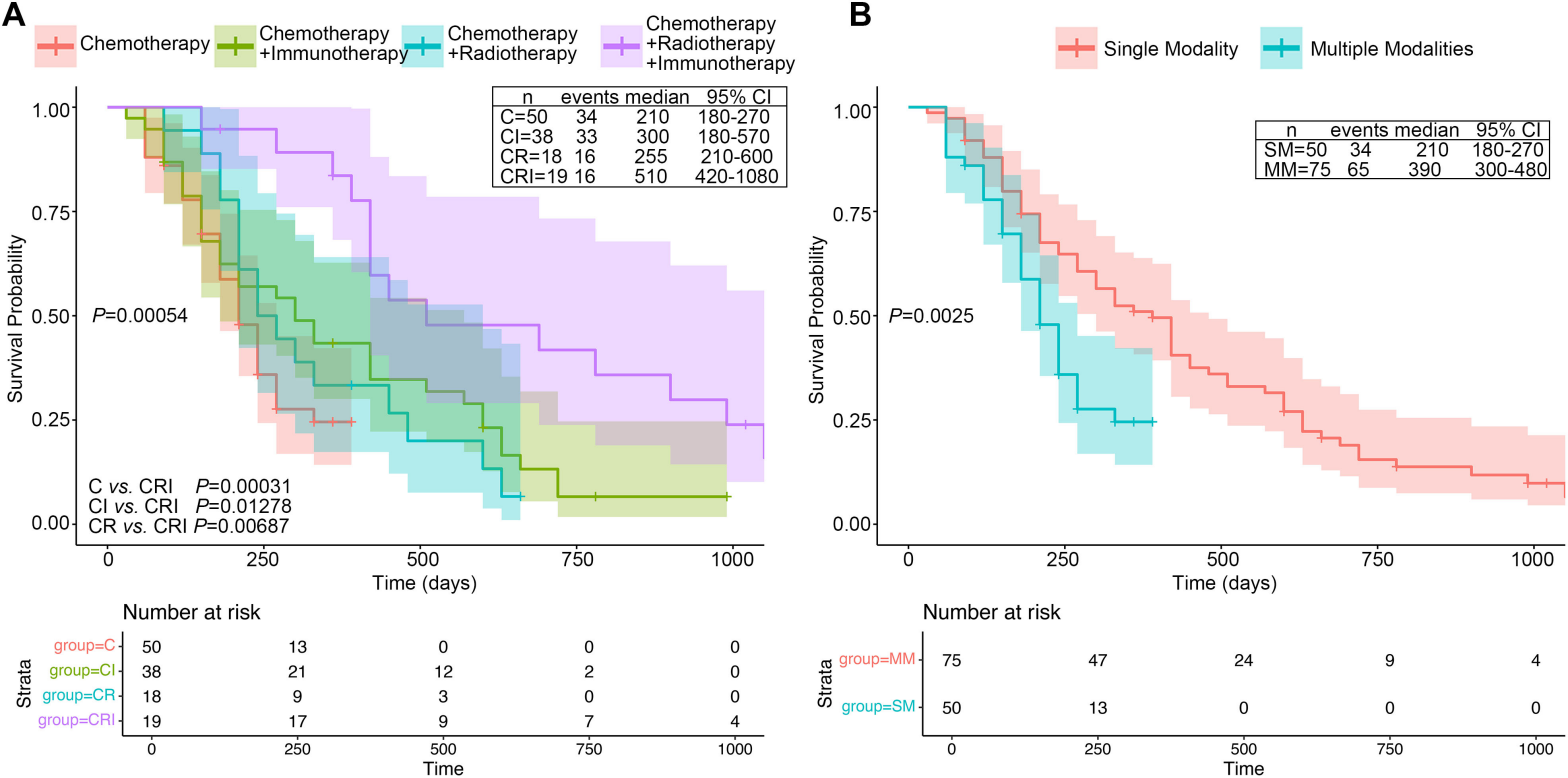
KM plots four different treatment groups in PC patients. **(A)** KM curve of OS in the four groups among C (Chemotherapy), CI (Chemotherapy plus Immunotherapy), CR (Chemotherapy plus Radiotherapy) and CRI (trial modal therapy). **(B)** KM curve of OS between monomodal and multimodal groups. Median survival time and confidence interval distribution among different treatment modalities groups were labeled alongside the KM curves.

A total of 125 pancreatic cancer patients were included in this study based on the predefined inclusion and exclusion criteria, with 32 patients surviving. The patients were divided into four treatment modality groups and the survival time among four groups were compared. Initially, an univariate Cox regression analysis (Table 2) indicated that treatment modality could serve as a protective factor (HR = 0.48, 95% CI = 0.30–0.78, *P* = 0.0027), suggesting that multimodality could significantly improve the prognosis of patients with late-stage pancreatic cancer. Kaplan–Meier (KM) plot indicated that combined treatment modality was associated with better survival in advanced PC patient (*P* = 0.00054). The median survival time of the four groups was: CRI = 510 days, CI = 300 days, CR = 255 days, and C = 210 days, respectively (Figure 2A). For multiple comparisons, the RIC group had significantly better prognosis than other groups (CRI vs. CI: *P* = 0.0128; CRI vs. CR: *P* = 0.0069; CRI vs. C: *P* = 0.0003). Next, we combined CR, CI and CRI as multimodal treatment groups. In comparison with monomodal treatment group (chemotherapy alone), multimodal treatment could significantly extend the survival of PC patients (*P* < 0.0025) (Figure 2B). Thus, we found that the prognosis of the CRI group was better than the other three treatment methods, with the longest median survival time and a statistically significant difference.

**TABLE 2:** Univariate Cox analysis.

Variables	HR	95% CI	*P*
Modality	0.4835	0.3006–0.7775	0.0027
Age	1.010	0.9890–1.031	0.3553
Gender	1.080	0.7243–1.609	0.7067
Histology	0.6389	0.4268–0.9563	0.02946
IL2	0.6192	0.5169–0.7416	1.92 × 10^−7^
IL4	0.8864	0.8270–0.9500	0.0007
IFN-γ	0.9830	0.9728–0.9932	0.0011
IL6	1.001	1.001–1.002	0.0001
IL17	1.034	1.026–1.042	3.61 × 10^−18^
NLR	14.61	3.116–68.46	0.0007
LDH	1.003	1.002–1.004	6.21 × 10^–10^
CEA	1.000	0.9987–1.002	0.7743
AFP	0.9799	0.8806–1.090	0.7087
CA19-9	1.000	0.99996–1.000	0.8838
β2-MG	0.9632	0.8536–1.087	0.5433

### 3.2 Multivariate Cox analysis of clinical meaningful variables affecting the prognosis of PC patients

**FIGURE 3:**
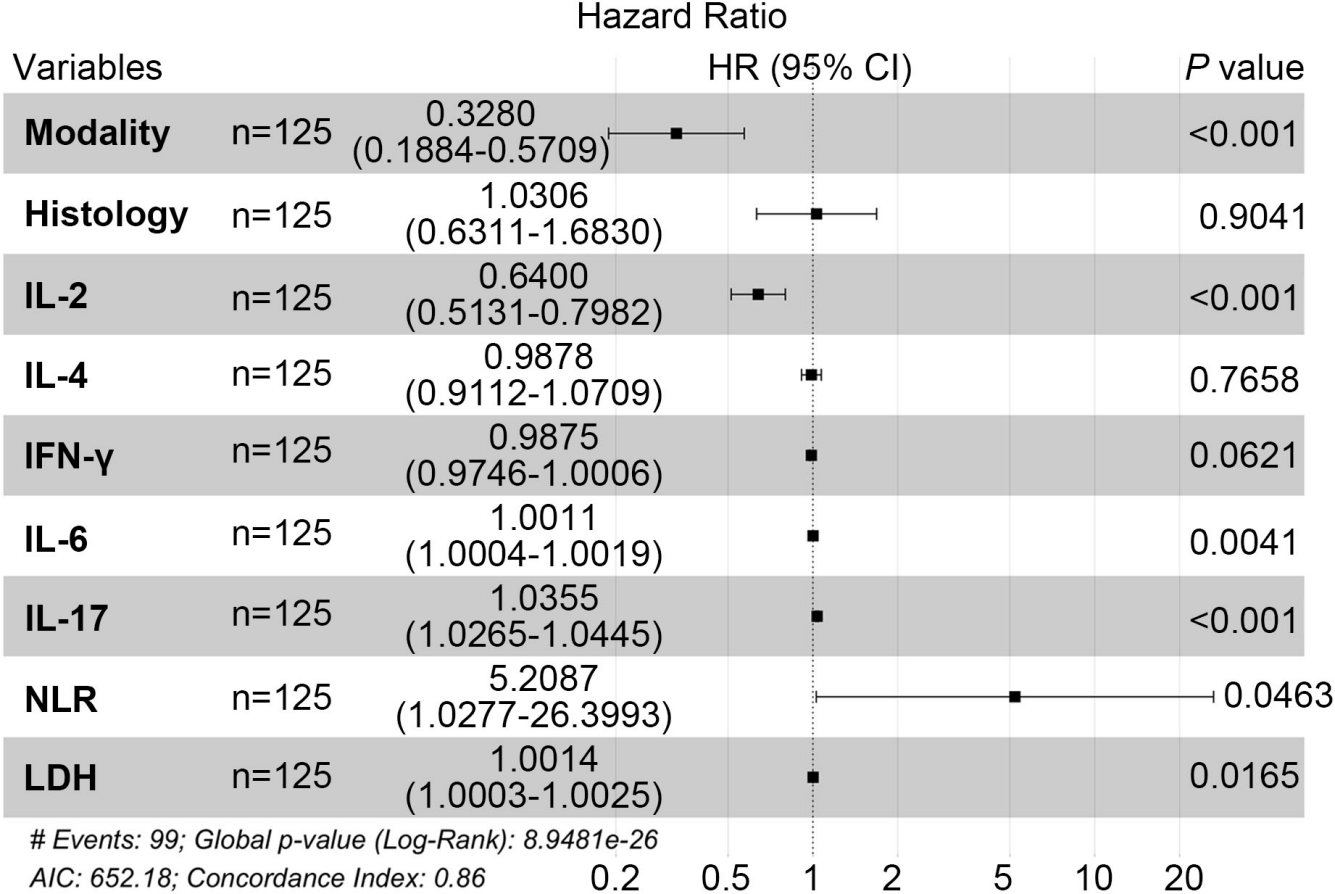
Multi-factor random forest diagram in PC patients.

Initially, we reviewed the clinical data of patients, conducted univariate analysis, and combined with literature research on prognostic factors of pancreatic cancer, finally including 11 biological indicators (IL-2, IL-4, IL-6, IL-17, IFN-γ, NLR, LDH, CEA, AFP, CA19-9, β2-MG). The univariate Cox regression analysis indicated that histology, IL-2, IL-4, IFN-γ, IL-6, IL-17, NLR, LDH were associated with prognosis of PC (*P* < 0.05) (Table 2). Next, we conducted a multivariate Cox analysis to identify independent factors associated with prognosis of PC. In addition to treatment modality, forest plot demonstrated that five variables, IL-2, IL-6, IL-17, NLR and LDH were screened with statistical significance (*P* < 0.05) (Figure 3). Moreover, IL-2 was prognostic protective factor (HR = 0.64, *P* < 0.01), while NLR was prognostic risk factor (HR = 5.21, *P* < 0.05).

### 3.3 Machine learning model construction and evaluation for binary classification tasks

Based on the multivariate Cox analysis of the treatment mode, a total of five metrics were included in this study, including three immunological metrics (L-2, IL-6, IL-17) and three clinical biochemical parameters (NLR, LDH). All variables were standardized before model training to improve model convergence efficiency. Four machine learning models (LR, SVM, RF, and XGBoost) were employed to establish the corresponding prognostic prediction models for binary classification and compare the differences in the classification performance of different machine learning models for unimodal versus bimodal plus trimodal therapy. All the model performance parameters were summarized in Table 3. As a result, ensemble methods based on tree models demonstrated a clear advantage in classification performance. Overall, XGBoost model achieved the best overall discriminative ability on the test set, with an AUC of 0.783 and an accuracy of 78.9%. It exhibited particularly strong performance in specificity (85.7%) and precision (81.8%), indicating its high reliability in identifying patients receiving single-modality treatment while effectively controlling false positives. Meanwhile, the Random Forest model achieved the highest sensitivity (82.6%), showing superior ability to identify multi-modality treatment patients—an important feature for minimizing missed diagnoses in clinical practice. Both ensemble models yielded F1 scores above 80%, reflecting a favorable balance between precision and recall.

**TABLE 3:** Model performance on the test set.

Models	LR	SVM	RF	XGboost
AUC	0.800	0.722	0.771	0.783
Accuracy	0.658	0.632	0.763	0.789
Specificity	0.600	0.400	0.792	0.857
Sensitivity (Recall)	0.696	0.783	0.826	0.783
Precision	0.727	0.667	0.792	0.818
F1 Score	0.711	0.720	0.809	0.800
Average precision (AP)	0.853	0.830	0.848	0.857

**FIGURE 4:**
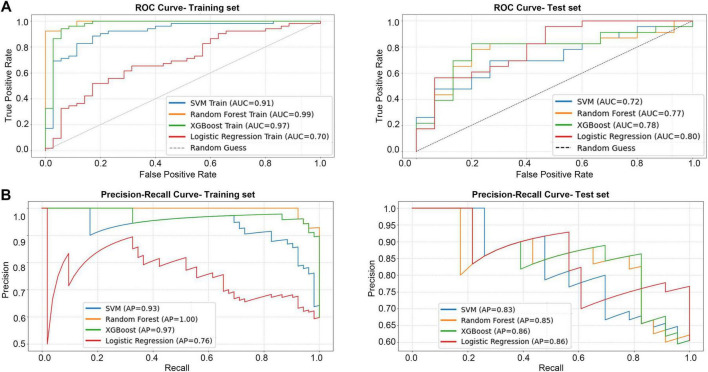
The performance of four models in the binary classification task. **(A)** ROC curve for training set and test set. **(B)** PR curve for training set and test set.

A particularly noteworthy aspect of this experiment is the insight into model generalization ability, revealed by the comparison between training and testing performance. As shown in Figure 4, RF and XGBoost achieved exceptionally high AUCs of 0.99 and 0.97 during model training, indicating strong fitting capability. On the test set, their AUCs decreased to 0.77 and 0.78, yet both maintained acceptable discriminative power. Precision-recall curve analysis further demonstrated that XGBoost attained both high AUC (0.78) and average precision (0.86) on the test data, achieving the optimal balance between discrimination and classification accuracy, thus highlighting its potential as a clinical predictive tool.

In contrast, LR, although showing relatively stable generalization (AUC = 0.80), exhibited limited classification efficiency (accuracy 65.79%, F1 = 71.11%), underscoring the inherent limitations of linear models in capturing complex non-linear relationships. The SVM performed the worst overall, and its combination of high sensitivity (78.26%) and low specificity (40%) suggested a strong bias toward predicting multi-modality treatment, resulting in excessive false positives and restricting its clinical applicability.

**FIGURE 5:**
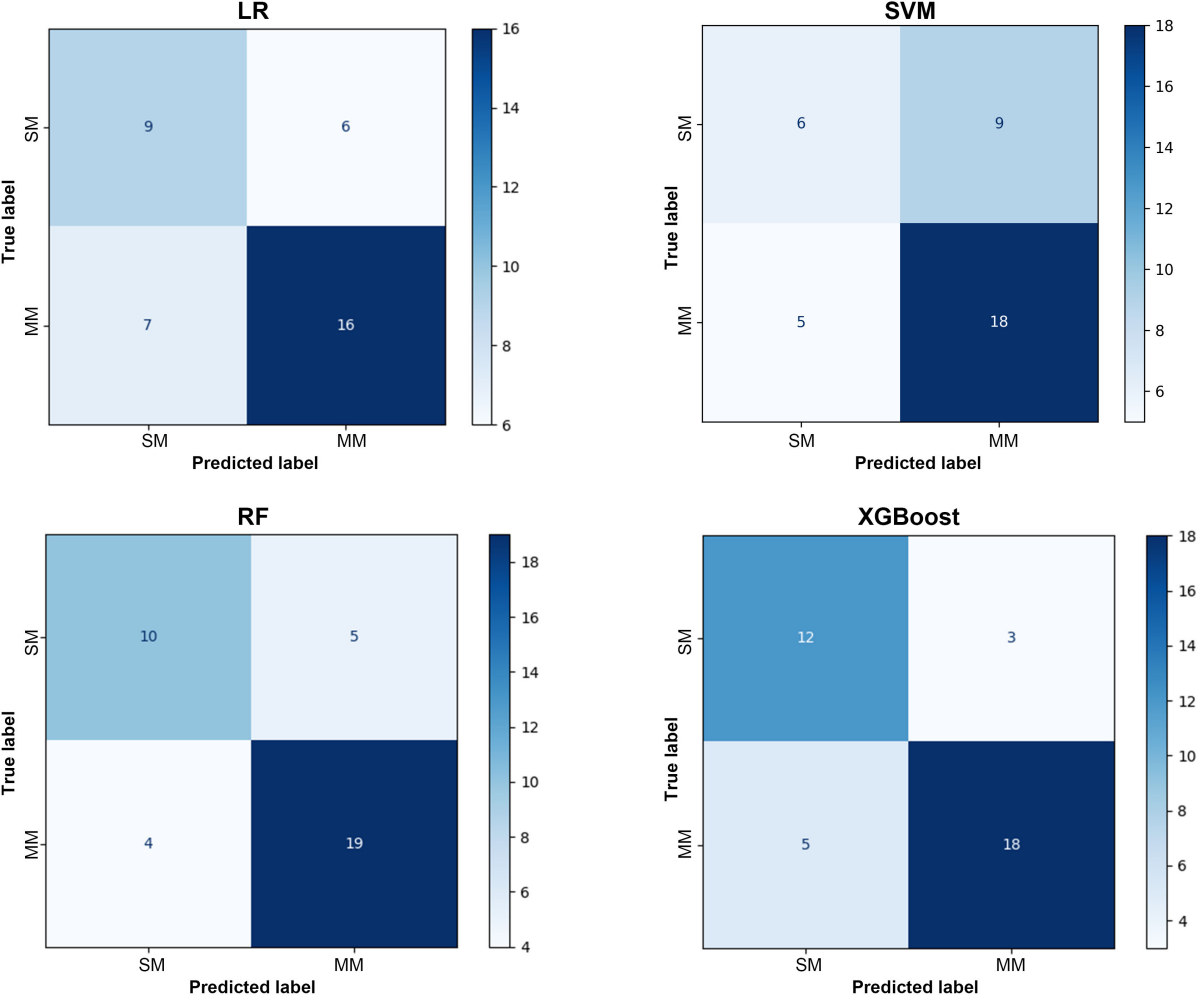
Confusion matrix of the test set.

As illustrated in Figure 5, the confusion matrix analysis further supports these findings. The RF model demonstrated the most balanced classification behavior, maintaining high true positive and true negative rates with a notably lower misclassification rate. The performance of the XGBoost model was comparable, achieving high specificity without compromising sensitivity. These results are consistent with the prior metric analyses, confirming the robustness and reliability of tree-based ensemble algorithms in handling complex clinical data. The above results indicated that the RF and XGBoost models, which were based on decision tree integration, showed significant advantages in dealing with high dimensional and unbalanced clinical data. They were especially effective in striking a better balance between sensitivity and specificity, which was significantly better than that of the traditional LR and SVM models.

### 3.4 Explanatory nature of model parameters

**FIGURE 6:**
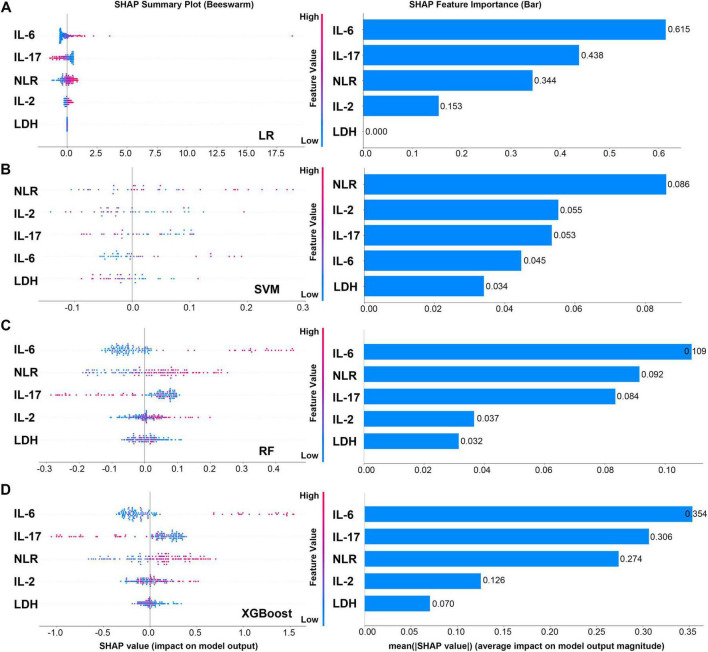
SHAP plots and feature importance bar charts of four models. **(A)** SHAP plots and feature importance bar charts of LR model. **(B)** SHAP plots and feature importance bar charts of SVM model. **(C)** SHAP plots and feature importance bar charts of RF model. **(D)** SHAP plots and feature importance bar charts of XGboost model.

To systematically evaluate the relative importance of each clinical feature in predicting the treatment mode (SM vs. MM) of pancreatic cancer, we conducted an explanatory analysis of four machine learning models using SHAP method. There are some differences in the feature contribution ranking of each model, but it also reveals key consistency rules. SHAP plots revealed that the proinflammatory cytokine IL-6 was dominant in most models. Among the three models with good discriminant performance, LR, RF and XGBoost, IL-6 was identified as the predictor with the highest contribution, and its average SHAP value was particularly prominent in the LR model (0.615). This agreement across models suggests that the systemic inflammatory response represented by IL-6 is a stable and robust biological feature that distinguishes treatment modalities in patients (Figure 6).

IL-17 and NLR are important auxiliary discriminant indicators, and their importance is model-dependent. IL-17 remained in the top three features in LR, RF and XGBoost models, which confirmed its key role in the tumor immune microenvironment. NLR became the primary feature in the SVM model, and made a significant contribution in LR and RF models, suggesting that systemic inflammatory burden is also a prediction dimension that cannot be ignored.

It is worth noting that the decision logic of different algorithms differs significantly. The XGBoost and RF models with the best performance both gave higher weights to IL-6 and IL-17, and the decision logic was clear and consistent with biological cognition. In contrast, the SVM model generally has a low absolute value of feature importance (the highest NLR is only 0.086), and its ranking (NLR > IL-2 > IL-17 > IL-6) is quite different from other models, which echoes its relatively low discriminative performance in this task. This suggests that it may not effectively capture the most important prognostic signal in the data.

Furthermore, LDH was consistently judged to be the least contributing feature across all models, indicating that it provides much less predictive information than specific immune inflammatory indicators in this specific treatment mode discrimination task. Altogether, in the binary classification tasks performed using the four machine learning models, IL-6 and IL-17 are the most critical biomarkers driving the decision of prognosis prediction models.

## 4 Discussion

To date, finding effective biomarkers to objectively assess the prognosis of pancreatic cancer patients remains a prominent issue in clinical research (26, 27). Existing markers commonly used for screening and diagnosing pancreatic cancer, such as CA19-9, suffer from insufficient specificity and sensitivity in early diagnosis (21, 28). This limitation leads to many patients being diagnosed at middle or late stages of the disease. Therefore, there is an urgent need for new prognostic markers to address this deficiency. Based on the established inclusion and exclusion criteria, a total of 125 PC patients were enrolled in this study. Their inflammation-related indexes, tumor markers, and serum biochemistry at the time of their first treatment in our hospital were collected. Cross-sectional comparative analysis revealed that prognosis improved with more comprehensive treatment modalities. Previous studies indicate that the combined application of various anti-pancreatic cancer treatment modes can synergistically enhance therapeutic effects and improve patient prognosis through different mechanisms, but there was still no detailed research on prognostic markers for various treatment modalities. Our findings may have important clinical implications for identifying patients who could benefit from chemotherapy alone or from model-guided therapy, and for elucidating the underlying molecular mechanisms.

Machine learning models can process large amounts of high-dimensional clinical data to provide personalized prognostic assessments for patients. In this study, four machine learning algorithms were used to construct predictive models based on the survival times of pancreatic cancer patients undergoing four different treatment modalities. The goal was to improve predictive accuracy and reliability and guide clinical practice. From the perspective of algorithmic characteristics, the superior performance of tree-based ensemble models likely arises from their strong capacity to capture complex feature interactions. In contrast, traditional models, constrained by their linear nature, failed to fully exploit the intricate patterns within the data, resulting in inferior performance. The high specificity of the XGBoost model makes it particularly suitable for clinical scenarios where false positives must be strictly controlled, such as precision medical resource allocation. Conversely, the high sensitivity of the Random Forest model provides distinct advantages in disease screening and early intervention. However, the performance gap between training and testing phases observed in ensemble methods underscores the necessity of monitoring model complexity and data suitability to prevent overfitting in clinical applications.

SHAP analysis revealed that inflammatory cytokines such as IL-6 and IL-17 contributed most significantly in tree models—a finding consistent with known biological mechanisms—thereby validating their ability to identify key prognostic biomarkers. Our finding indicates that specific inflammatory pathways in the tumor microenvironment, rather than general tumor burden indicators, are the core biological basis for differentiating the treatment patterns of pancreatic cancer patients, which provides an important theoretical basis for the development of individualized In conclusion, this comprehensive multi-dimensional evaluation confirms that tree-based ensemble learning methods possess significant advantages in pancreatic cancer treatment mode classification tasks. Among them, XGBoost demonstrates the best overall performance and strongest potential for clinical translation. These findings provide a reliable algorithmic foundation for clinical prognosis prediction and lay the groundwork for developing individualized treatment decision-support systems. Future research should focus on expanding the dataset, refining feature engineering, and validating model effectiveness and practicality in real-world clinical settings.

It is well documented that chemotherapy and radiotherapy can improve the effectiveness of immunotherapy by increasing tumor antigen expression and stimulating anti-tumor immune responses. Chemotherapy and radiotherapy, traditionally regarded as cytotoxic treatments, are now recognized as potent inducers of immunogenic cell death (ICD), leading to the release of tumor-associated antigens and neoantigens. Consequently, the tumor microenvironment may shift from an immunosuppressive “cold” state to a more active inflamed “hot,” making it more susceptible to immune-mediated attack (29). Accumulating evidence suggests inflammatory cytokines can either promote or inhibit tumor progression depending on the cancer context, influencing processes like angiogenesis, proliferation, and immunosuppression (30). Circulating cytokines derived from the tumor microenvironment reflect tumor-associated inflammatory activity and can serve as minimally invasive biomarkers for cancer detection and prognosis (31). Serum IL-17 levels affect prognosis by influencing the tumor microenvironment (32). Studies showed that the combined use of anti-IL-17A antibody and gemcitabine can induce M1 polarization of macrophages and enhance anti-tumor response (33). Therefore, blockade of IL-17 has been shown to modulate the immunosuppressive tumor microenvironment and IL-17 may act as a determinant of differential response between chemotherapy alone and bimodal treatment strategies combined with chemotherapy and immunotherapy.

However, this study has some limitations. As a single-center retrospective study, it included a relatively small number of eligible pancreatic cancer patients. Future work should increase the sample size to revalidate the conclusions drawn in this study.

## 5 Conclusion

In conclusion, our study constructed a practical tool to assist in prognostic determination for pancreatic cancer patients receiving different treatment modalities, based on routine clinical laboratory indices using an artificial intelligence approach. This comprehensive multi-dimensional evaluation confirms that tree-based ensemble-learning methods possess significant advantages in pancreatic cancer treatment mode classification tasks. Among them, XGBoost demonstrates the best overall performance and strongest potential for clinical translation. These findings provide a reliable algorithmic foundation for clinical prognosis prediction and lay the groundwork for developing individualized treatment decision-support systems. Future research should focus on expanding the dataset, refining feature engineering, and validating model effectiveness and practicality in real-world clinical settings. This tool can provide personalized prognostic assessments for each patient. During treatment, clinicians can use this predictive model to make more rational decisions and adjust treatment plans to achieve better outcomes.

## Data Availability

The raw data supporting the conclusions of this article will be made available by the corresponding authors.
